# Neurological update: dizziness

**DOI:** 10.1007/s00415-020-09748-w

**Published:** 2020-03-04

**Authors:** Diego Kaski

**Affiliations:** 1 Department of Neurology, National Hospital for Neurology and Neurosurgery, Queen Square, London, WC1N 3BG UK; 2 Centre for Vestibular and Behavioural Neurosciences, Department of Clinical and Motor Neurosciences, University College London, 33 Queen Square, London, WC1N 3BG UK

**Keywords:** Dizziness, Vestibular, Small vessel disease cerebral, Vestibular migraine, Persistent postural perceptual dizziness (PPPD)

## Abstract

The diagnosis and management of vertigo remains a challenge for clinicians, including general neurology. In recent years there have been advances in the understanding of established vestibular syndromes, and the development of treatments for existing vestibular diagnoses. In this ‘update’ I will review how our understanding of previously “unexplained” dizziness in the elderly is changing, explore novel insights into the pathophysiology of vestibular migraine, and its relationship to the newly coined term ‘persistent postural perceptual dizziness’, and finally discuss how a simple bedside oculomotor assessment may help identify vestibular presentations of stroke.

## Introduction

The world of dizziness has experienced a dramatic change over the last 3 decades, as new treatable syndromes have been identified, and novel treatments developed for existing vestibular diagnoses. Despite such progress, many clinicians, including neurologists, admit to a lack of confidence in the diagnosis and management of the dizzy patient, leading to circuitous patient journeys, from one specialty to another. Most emergency and primary care ‘dizzy’ referrals in the UK are fielded to ENT surgeons, a departure from *neuro* towards *otology*, although it could be argued that vertigo is a neurological symptom, a cortically driven percept, irrespective of the causative insult.

One common challenge in the field is elderly patients reporting a vague sense of dizziness and imbalance, who as a result of normal audiovestibular testing, remain “unexplained”. I will review recent evidence suggesting possible mechanisms relating to small vessel disease that may contribute to this syndrome. Whilst new variants of benign paroxysmal positional vertigo (BPPV) have been recently described [1], the Epley and Semont treatment manoeuvres for the commonest type of BPPV are still not universally employed by neurologists [2], and BPPV remains under-diagnosed, and under-treated. The commonest differential diagnosis for BPPV is vestibular migraine, a condition that is increasingly recognised outside specialist centres, but remains under-diagnosed. Here, I review the most recent advances in vestibular migraine (VM) diagnosis and treatment. VM in turn is a common precursor to a more chronic form of dizziness recently renamed persistent postural perceptual dizziness (PPPD), and there has been a growth in the unravelling of the neurobiology of this disorder. Finally, vestibular neurology is rich in clinical bedside skills; indeed, an evaluation of eye movements may more precisely identify and localise a stroke than state-of-the-art imaging [3]. I describe and review the use and utility of the HINTS examination in stroke.

## “Unexplained” dizziness in the elderly

The symptom complex of subjective unsteadiness and a persistent sensation of light-headedness, without any rotational vertigo—that patients refer to as a vague sense of “dizziness”—is often reported by elderly patients attending neurology or balance clinics [4]. In light of a normal extensive battery of neurological and neuro-otological assessments, the patient’s dizziness may be termed “unexplained”, a particularly common and challenging problem in the elderly.

Cerebral small vessel disease (cSVD) is associated with a range of radiological findings, including white matter hyperintensities in the cerebral white matter on proton density-weighted, T2-weighted and fluid attenuated inversion recovery (FLAIR) MR images, considered to be vascular in origin [5, 6]. cSVD is a common finding in the ageing population, present in 10% of patients in their 70s and increases to 85% in the 90s [7]. Despite the established association between cSVD and gait disturbance, most large-scale studies of cSVD have, perhaps surprisingly, not reported on the presence (or absence) of dizziness symptoms in these patients. A prospective study of 26 older patients (> 75 years of age) with disequilibrium of unknown cause, found that in 12 cases they had a degree of MRI cSVD that correlated with poorer performance on objective measures of balance and frequency of falls [8]. In a subsequent longitudinal study with yearly examinations in 59 older subjects, Baloh et al. again found a higher correlation between white matter hyperintensities on MRI and changes in stance and gait [9].

Ahmad et al. sub-divided patients with cSVD into either ‘high’ or ‘low’ cSVD burden and identified that over 80% patients with high grades of cSVD suffered from otherwise unexplained dizziness, compared to 48% of patients with low-grade cSVD [4].The implication is that cSVD may be an independent factor in the development of balance and dizziness symptoms in the elderly.

**Fig. 1:**
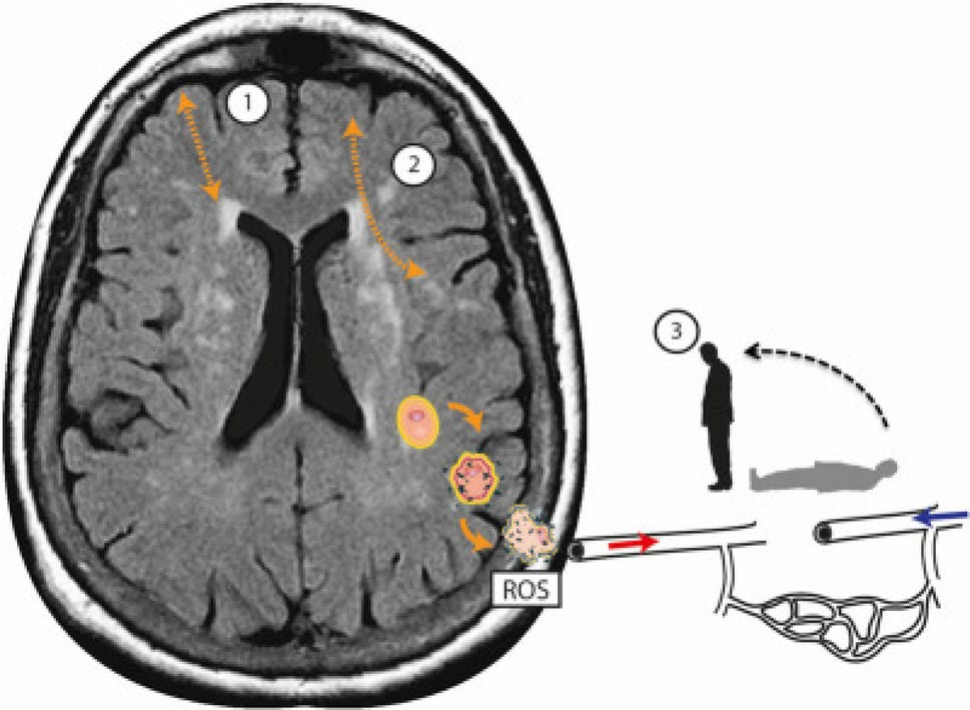
Schematic representation of the additional deleterious effects of postural blood pressure hypotension in cerebral small vessel disease associated dizziness in addition to cortico-subcortical (1), and cortico-cortical (2) disconnectivity. Localized oxidative stress processes damage the cerebral vasculature, leading to endothelial dysfunction and promoting neurodegenerative alterations in the brain tissue through reactive oxidative species (ROS). Microvascular endothelial dysfunction in turn disrupts cerebral autoregulation—which in healthy states maintains adequate and stable cerebral blood flow when blood pressure drops (3). This leads to intra-cerebral orthostatic hypotension and perfusion, manifest as postural light-headedness and imbalance. From Kaski et al. [10] with permission

Recently we proposed a theoretic framework for “unexplained” dizziness in the elderly whereby a central disturbance of vestibular processing and integration results from cSVD-related cortico-subcortical and cortico-cortical dysfunction (Fig. 1) [10]. In addition, hemodynamic changes in the blood flow within the white matter due to small vessel disease, in combination with cerebral postural hypotension, has been hypothesised to contribute to the postural lightheadedness in this condition (Fig. 1) [10]. This has been corroborated by electroencephalographic changes during postural challenges between elderly individuals with and without “unexplained” dizziness, partly driven by the presence of cSVD (Ibitoye R et al. manuscript in preparation). Further work is underway to explore the mechanisms by which cSVD may contribute to postural instability in the elderly, perhaps helping to identify novel treatment avenues targeting cortical excitability [11].

## Vestibular migraine

As arguably the most common neurological disorder, migraine affects approximately 15% of the general population [12], and although dizziness is also a common symptom in the general population [13], several studies have found a close association between dizziness and migraine beyond what one might expect through chance [14–16], suggesting a pathological link between migraine and dizziness [17]. Vestibular migraine (VM) is perhaps less-well recognised outside specialist vestibular clinics [18]. Indeed, although VM has recently been included in the third edition of the International Classification of Headache Disorders (ICHD-3) (Box 1), it is currently listed as an appendix [19], indicating the need for further validation with high-level evidence, which may lead to its formal acceptance in future revisions.

**Box 1:** Bárány Society diagnostic criteria for persistent postural perceptual dizziness

(A) One or more symptoms of dizziness, unsteadiness or non-spinning vertigo on most days for at least 3 months
Symptoms last for prolonged (hours-long) periods of time, but may wax and wane in severity
Symptoms need not be present continuously throughout the entire day
(B) Persistent symptoms occur without specific provocation, but are exacerbated by three factors: upright posture, active or passive motion without regard to direction or position, and exposure to moving visual stimuli or complex visual patterns
(C) The disorder is triggered by events that cause vertigo, unsteadiness, dizziness, or problems with balance, including acute, episodic or chronic vestibular syndromes, other neurological or medical illnesses, and psychological distress
When triggered by an acute or episodic precipitant, symptoms settle into the pattern of criterion A as the precipitant resolves, but may occur intermittently at first, and then consolidate into a persistent course
When triggered by a chronic precipitant, symptoms may develop slowly at first and worsen gradually
(D) Symptoms cause significant distress or functional impairment
(E) Symptoms are not better accounted for by another disease or disorder

Patients with VM experience episodic vestibular symptoms and migrainous features (nausea, photophobia, phonophobia, motion intolerance, catamenial association), with or without concurrent headache. They frequently report sensitivity to head motion and visual surroundings or disabling misperceptions such as a sudden imbalance or body tilt [20]. Clinical pearls that suggest VM include a pre-existing history of motion sickness, and particularly being unable to read in the passenger seat of a car due to nausea, and a discomfort during upgaze with the eye movement assessment. There may be central eye movement signs during an acute episode [21], but the examination is typically normal inter-ictally [19].

At a molecular level, there appears to a differential proinflammatory signature (namely IL-1β, CCL3, CCL22, and CXCL1 levels) capable of differentiating VM patients from patients with Meniere’s Disease, conditions that can overlap clinically [22]. Whether such pro-inflammatory markers are capable of segregating typical migraine from VM remains to be seen.

Neurophysiological findings do however suggest a specific abnormality of visuo-vestibular interaction as a pathophysiological mechanism in VM; for example, patients with VM have a longer duration of post-rotatory nystagmus compared with healthy controls or migraine patients without dizziness [23]. We have identified abnormally elevated reflexive and perceptual vestibular thresholds at baseline in vestibular migraine patients [24]. Following visual motion exposure, both reflex and perceptual thresholds were further increased in vestibular migraine patients relative to healthy controls, migraineurs without vestibular symptoms and patients with benign paroxysmal positional vertigo [24]. This supports the notion of specific altered visuo-vestibular cortical interactions in vestibular migraine. This notion is further supported by imaging studies in VM patients revealing structural and functional changes within the temporo-parietal regions [25, 26]-areas involved in sensory integration for coherent spatial perception [27, 28].

Further molecular, imaging, and neurophysiological studies are needed to better define the pathogenic signatures of vestibular migraine. Better understanding of symptoms related to visuo-vestibular integration in VM, such as tilt sensitivity, upright misperception [29], and ictal nystagmus [21] may provide further pathophysiological clues to this condition.

As for other types of migraine, both abortive and preventive medications have been used in VM patients. However, large-scale randomized placebo-controlled clinical trials in VM are missing, although a prospective randomized non-placebo-controlled study in VM patients suggested flunarizine is effective in decreasing the severity and frequency of vertigo attacks [30]. Table 1 summarises studies on preventive treatments for vestibular migraine. Life-style adjustment, trigger avoidance and vestibular rehabilitation are also shown to be beneficial in VM patients, where secondary psychological factors may also play a role in disease burden.

**Table 1:** Summary of studies on preventive treatments for vestibular migraine defined based on the ICHD-3 criteria. (from [20])

Drug	Daily dose	Study design	Duration	Outcome	
Propranolol*	40–160 mg	33 patients, prospective, randomized, controlled	4 months	Severity: DHI 55.8 ± 2.7 to 31.3 ± 3.7* VSS 7.3 ± 0.3 to 2.1 ± 0.4* Attacks per month: 12.6 ± 1.8 to 1.9 ± 0.7	Salviz et al. [31] (158)
Venlafaxine	37.5–150 mg	31 patients, prospective, randomized, controlled	4 months	Severity: DHI 50.9 ± 2.5 to 19.9 ± 2.9* VSS 7.1 ± 0.3 to 1.8 ± 0.5* Attacks per month: 12.2 ± 1.8 to 2.6 ± 1.1 *	Salviz et al. [31] (158)
Venlafaxine	37.5 mg	25 patients, prospective	3 months	Severity: DHI 41.7 ± 16.9 to 31.3 ± 14.1* VSS 5.9 ± 1.7 to 3.8 ± 1.2*	Liu et al. [32] (157)
Flunarizine	10 mg	25 patients, prospective	3 months	Severity: DHI 46.6 ± 15.1 to 39.8 ± 16.3* VSS 6.4 ± 1 .9 to 5.9 ± 1.6*	Liu et al. [32] (157)
Valproic acid	1000 mg	25 patients, prospective	3 months	Severity: DHI 46.8 ± 13.5 to 38.7 ± 13.6* VSS 5.8 ± 1.8 to 5.3 ± 1.0	Liu et al. [32] (157)
Cinnarizine	75 mg	24 patients, retrospective open-label	3 months	Attacks per month: 3.8 ± 1.1 to 0.4 ± 0.6*	Taghdiri et al. [33] (160)

*DHI* Dizziness handicap inventory, *VSS* Vertigo Severity score (Liu et al.)

*p < 0.05

## Persistent postural perceptual dizziness

Persistent postural perceptual dizziness is characterised by persistent dizziness and perceived instability, worse in the upright position and in busy visual environments [34]. An acute episode of dizziness may precede these symptoms, and the disorder will usually emerge with resolution of the triggering event. The most common precipitants are central or peripheral vestibular disorders such as BPPV (25%) and episodes of vestibular migraine (VM) (20%), with head trauma, panic attack and generalised anxiety disorders each accounting for a further 15% [34].

The Bárány Society have set out diagnostic criteria (Box 1) [35] reliant upon a thorough clinical history. Like many other functional neurological symptoms, PPPD is not a diagnosis of exclusion, although physical, neurophysiological, biochemical and radiological investigation may be required to fully explore alternative differentials. The clinician must be mindful that PPPD can co-occur with structural vestibular and other neurological disorders. For example, a patient with VM may develop PPPD with acute vertiginous episodes (VM) on a background of persistent dizziness (PPPD) [36].

Whilst the pathophysiology of functional vestibular disorders such as PPPD is not yet clear, normal physiological and behavioural responses to an acute postural threat appear to become inappropriately sustained after remission of the acute event [34]. Patients who develop PPPD after an acute event show persistent high visual dependence (an over-reliance on vision for balance), high anxiety and hypervigilance to balance sensations compared with those who recover well after an acute vestibular insult [37]. Prior anxiety and neurotic personality (state and trait anxiety) appear to predispose to this maladaptation [38].

Maladaptive behaviours in PPPD likely relate to heightened sensitivity to minor discrepancies between anticipated and actual afferent postural signals. This results in greater attention and effort to actively maintain balance, in turn feeding hypervigilance and reliance on ‘high-risk’ strategies. The misperceived discrepancy between predicted and actual risk becomes reinforced and a vicious cycle is established, leading to a ‘scaling mismatch’ between actual and perceived postural movements. Normal balance can be restored temporarily with distraction (e.g. performing a cognitive task during Romberg stance), which can therefore be used therapeutically in rehabilitation [34].

Neuroimaging studies in turn have demonstrated that activity and connectivity in brain regions that process visual, vestibular and spatial information differ between individuals with and without PPPD. A failure of top-down cortical network suppression of ascending postural information may result in persistence of the acute, high-risk postural behaviour [38].

Whether a top-down, or bottom-up disorder, from a practical perspective, it is important to explain to patients that PPPD is a common cause of chronic dizziness that can be treated successfully. It may also be valuable to demonstrate the reversibility of some symptoms using distraction techniques during physical examination. Treatment options include: (1) vestibular and balance physiotherapy, with the aim of reducing visual dependence and desensitising the balance system, (2) medications, including Amitriptyline and Sertraline [39], and (3) cognitive behavioural therapy (CBT) to reduce hypervigilance and anxiety, and related behaviours [40]. Combination of these three interventions using a “cognitive physiotherapy” approach is recommended, at the earliest possible stage.

## Dizziness and stroke

Identifying stroke in a patient with acute vertigo is challenging particularly in the absence of accompanying neurological symptoms and signs. This is particularly true of strokes in the hyperacute phase and small strokes that may escape detection on imaging [3]. Small strokes causing isolated vertigo therefore carry a higher chance of misdiagnosis in the emergency setting, even more so as they escape the Face Arm Speech Time (FAST) stroke symptoms.

The HINTS-plus (Head Impulse, direction-changing Nystagmus, and a Test of Skew, plus a bedside assessment of hearing) examination can be used to help identify posterior circulation stroke in patients with prolonged acute vertigo and one or more risk factors for stroke. In one study, this is 100% sensitive and 96% specific for posterior circulation stroke [3]. Although the implementation of HINTS-plus evaluation in the ED may be valuable and feasible for neurologists, it poses a significant challenge for emergency physicians, with only a 9% take-up rate following a 2-month implementation program [41]. Thus, its value in the primary care setting is uncertain. Application of artificial intelligence and tele-consultation [42], incorporating a structured oculomotor assessment, and perhaps including vascular/perfusion imaging for isolated vestibular syndromes, may be future perspectives for real-time decision making in acute dizziness and vertigo.

## Conclusions

The field of vestibular science continues to make advances into the twenty-first century, with current priorities being the introduction and expansion of vestibular neurology into undergraduate medical curricula, to improve clinical diagnosis of patients with vertigo in acute, primary, and secondary care, encouragement of large-scale randomised controlled clinical trials for vestibular migraine, improved understanding of the mechanisms leading to chronic dizziness (PPPD), and exploration of how multiple factors, including cSVD, may contribute to otherwise “unexplained” dizziness in the elderly.
